# Gene diversity, agroecological structure and introgression patterns among village chicken populations across North, West and Central Africa

**DOI:** 10.1186/1471-2156-13-34

**Published:** 2012-05-07

**Authors:** Grégoire Leroy, Boniface B Kayang, Issaka AK Youssao, Chia V Yapi-Gnaoré, Richard Osei-Amponsah, N’Goran E Loukou, Jean-Claude Fotsa, Khalid Benabdeljelil, Bertrand Bed’hom, Michèle Tixier-Boichard, Xavier Rognon

**Affiliations:** 1 AgroParisTech, UMR1313 Génétique Animale et Biologie Intégrative, Paris 05, F-75231, France; 2 INRA, UMR1313 Génétique Animale et Biologie Intégrative, Jouy-en-Josas, 78352, France; 3 University of Ghana, Legon, Ghana; 4 Université d’Abomey-Calavi, Ecole Polytechnique d’Abomey-Calavi, Cotonou, 01 BP 2009, Bénin; 5 Centre National de la Recherche Agronomique, Abidjan, 01 BP 1740, Côte d’Ivoire; 6 Université de Cocody, Abidjan, 22 BP 1244, Côte d’Ivoire; 7 Station Spécialisée de Recherche Agricole de Mankon (SRRAD), Bamenda, BP 4099, Cameroun; 8 Institut Agronomique et Vétérinaire Hassan II, DPBA, Rabat Instituts, 10101, Rabat, BP 6202, Maroc

## Abstract

**Background:**

Chickens represent an important animal genetic resource for improving farmers’ income in Africa. The present study provides a comparative analysis of the genetic diversity of village chickens across a subset of African countries. Four hundred seventy-two chickens were sampled in 23 administrative provinces across Cameroon, Benin, Ghana, Côte d’Ivoire, and Morocco. Geographical coordinates were recorded to analyze the relationships between geographic distribution and genetic diversity. Molecular characterization was performed with a set of 22 microsatellite markers. Five commercial lines, broilers and layers, were also genotyped to investigate potential gene flow. A genetic diversity analysis was conducted both within and between populations.

**Results:**

High heterozygosity levels, ranging from 0.51 to 0.67, were reported for all local populations, corresponding to the values usually found in scavenging populations worldwide. Allelic richness varied from 2.04 for a commercial line to 4.84 for one population from Côte d’Ivoire. Evidence of gene flow between commercial and local populations was observed in Morocco and in Cameroon, which could be related to long-term improvement programs with the distribution of crossbred chicks. The impact of such introgressions seemed rather limited, probably because of poor adaptation of exotic birds to village conditions, and because of the consumers’ preference for local chickens. No such gene flow was observed in Benin, Ghana, and Côte d’Ivoire, where improvement programs are also less developed. The clustering approach revealed an interesting similarity between local populations found in regions sharing high levels of precipitation, from Cameroon to Côte d’Ivoire. Restricting the study to Benin, Ghana, and Côte d’Ivoire, did not result in a typical breed structure but a south-west to north-east gradient was observed. Three genetically differentiated areas *(P* < 0.01) were identified, matching with Major Farming Systems (namely Tree Crop, Cereal-Root Crop, and Root Crop) described by the FAO.

**Conclusions:**

Local chickens form a highly variable gene pool constituting a valuable resource for human populations. Climatic conditions, farming systems, and cultural practices may influence the genetic diversity of village chickens in Africa. A higher density of markers would be needed to identify more precisely the relative importance of these factors.

## Background

Knowledge-based management of animal genetic resources (AnGR) is critical to answer the current agricultural, socio-economic, and environmental challenges. Consequently, characterization of AnGR constitutes one of the priorities of the FAO global plan of action for AnGR [1], in particular in developing countries, where there is a lack of information regarding what and how to conserve, develop, and select among local breeds.

Village poultry make a significant contribution to poverty alleviation and household food security in many developing countries [2]. About 1.5 billion chickens are raised in Africa, 80% of them belonging to local chicken populations [3]. Indigenous chickens are considered to make a significant contribution to food security and the economical sustainability of rural households [4-6]. However, little is known about their genetic diversity. A recent FAO survey has shown that economic drivers and poor livestock sector policies are the main threats to AnGR: intensification of agriculture, importation of exotic breeds, and indiscriminate cross-breeding [7]. In the case of poultry, poor conservation strategies represent a relatively important threat, and incentives for a continued and sustainable use of local populations are lacking. Conservation strategies require a good knowledge of the genetic structure of these local populations, within or between countries, as well as an assessment of their diversity at the molecular level, to provide recommendations regarding their future management. Several studies of the genetic diversity and structure of local chicken populations in Africa have been done separately for different countries [8-15], and very few have considered a larger region such as East Africa [16]. More specifically, possible relationships between genetic diversity and environmental conditions have been investigated for chicken populations with contrasted results depending on the country of study [9,11,16]. Thus, an integrated study encompassing several African countries is still lacking but is undoubtedly required in order to give a more complete analysis of the current diversity of local chickens on this continent, where domestic chickens arrived from various origins such as India and the Mediterranean area [17,18].

The aim of the present study was to provide a large-scale analysis of the genetic diversity of local chickens in several countries from the central, western and northern parts of the African continent, in order to address questions important for further conservation strategies. These questions deal with (i) the amount of genetic diversity found within these populations, (ii) the search for a possible correlation between the genetic structure and agroecological distribution, and (iii) the detection of a possible gene flow between local populations and commercial lines.

## Methods

### Sample collection and genotypes

**Figure 1:**
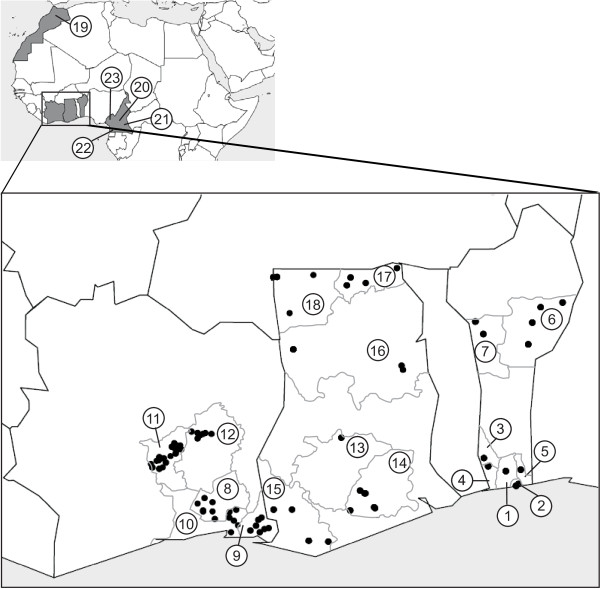
**Geographic location of the 23 local chicken populations sampled throughout Côte d’Ivoire, Ghana, Benin, Cameroon and Morocco.** Population units are numbered as indicated in Table 1. Dots represent collection sites for Benin, Ghana and Côte d’Ivoire.

The sampling design involved 5 countries (Benin, Côte d’Ivoire, Ghana, Cameroon, and Morocco). Blood samples were drawn from the wing vein of 472 local adult chickens. Samples representative of Benin, Côte d’Ivoire, and Ghana chicken populations were collected throughout each country (Table 1, Figure 1). These populations have been independently investigated in previous studies [12-14] respectively). For a given village investigated, a mean number of 2 households was randomly chosen, and, for a given household, 2 chickens were sampled on average (80% of females). The number of villages investigated for a given administrative region ranged from 2 to 21. Each sample was assigned geographical coordinates based upon the village positions. For Benin, samples from the northern area were obtained from the Borgou (N = 38) and Donga (N = 17) administrative regions, within a sampling zone lying between 9°13′–10°10′ N and 1°18′–3°13′ E. Samples from the southern area came from the Atlantic (N = 20), Couffo (N = 10), Littoral (N = 10), Mono (N = 10), and Ouémé (N = 8) regions, which lie between 6°12′–6°37′ N and 1°24′–2°23′ E. For Côte d’Ivoire, only the southern area was covered, including the Lacs (N = 41), N’Zi-Comoé (N = 22), Agnéby (N = 18), Sud-Comoé (N = 17), and Lagunes (N = 19) regions, between 5°20′–7°37′ N and 2°56′–5°27′ W. For Ghana, the northern area included the Northern (N = 25), Upper-West (N = 23), and Upper-East (N = 5) regions, between 8°50′–11°01′ N and 0°03′–2°51′ W, while the southern area included the Western (N = 32), Eastern (N = 21), and Ashanti (N = 6) regions, between 5°08′–7°22′ N and 0°39′–2°49′ W. Cameroonian samples (sex-ratio 80% of females) were obtained from local experimental stocks, reared at the Mankon Research Station, after incubating fertile eggs sampled from 93 villages distributed across four regions ([19]), namely the Centre (30 chicks), South (28 chicks), North-West/West (22 chicks), and East (5 chicks) regions lying between 2°50′–7° N and 10°–15° E. Moroccan samples (N = 45) were randomly collected in the Agoudim village (Meknes region, in the Middle Atlas; 30°50′ N–5°35′ W), on three remote sites spread over several kilometers. Within each site, 5 to 15 households were selected, 1 to 2 individuals were sampled for a given household (sex-ratio: 50/50). For comparative purposes and searching of potential gene flow, 5 commercial lines [20], including 3 broiler (N = 25–29) and 2 layer (N = 25) lines (Table 1), were also analyzed. These lines were chosen to be representative of the main commercial lines usually imported into these countries, such as: white egg layers (Hy-line, Lohmann), brown egg layers (Isa Brown) and broilers (Cobb, Hubbard, Ross).

**Table 1:** Origin and sample size of the 28 chicken populations

**Type**	**Country**	**Region**	**Code**	**Sample size**	**Location number**
*Local*	**Benin**	Atlantique	BEN-Atl	20	1
*populations*		Littoral	BEN-Lit	10	2
		Couffo	BEN-Cou	10	3
		Mono	BEN-Mon	10	4
		Ouémé	BEN-Oué	8	5
		Borgou	BEN-Bor	38	6
		Donga	BEN-Don	17	7
	**Côte**	Agnéby	CIV-Agn	18	8
	**d’Ivoire**	Sud-Comoé	CIV-SCo	17	9
		Lagunes	CIV-Lag	19	10
		Lacs	CIV-Lac	41	11
		N’Zi-Comoé	CIV-NCo	22	12
	**Ghana**	Ashanti	GHA-Ash	6	13
		Eastern	GHA-Eas	27	14
		Western	GHA-Wes	26	15
		Northern	GHA-Nor	25	16
		Upper-East	GHA-UEa	16	17
		Upper-West	GHA-UWe	12	18
	**Maroc**	Meknes	MAR-Mek	45	19
	**Cameroun**	Centre	CAM-Cen	30	20
		Est	CAM-Est	5	21
		Sud	CAM-Sud	28	22
		Ouest/Nord-Ouest	CAM-ONO	22	23
*Commercial lines*					
*Broiler*	Broiler-sire line-C		BS-C	25	
	Broiler-sire line-D		BS-D	29	
	Broiler-dam line-B		BD-B	25	
*Layer*	White egg layer-A		WEL-A	25	
	Brown egg layer-C		BEL-C	25	

Genomic DNA was extracted from blood using the Qiagen® Kit (Qiagen, Valencia, CA, USA) at the biotechnology laboratory of the University of Ghana (samples from Benin, Côte d’Ivoire, Ghana, and Cameroon) or using the NaOH extraction protocol at the IAV (Moroccan samples). A total of 601 individuals, including 472 local African chickens and 129 individuals from commercial lines, were genotyped for 22 microsatellite loci from the AvianDiv panel [21]. PCR amplification and genotyping were performed by the same laboratory (Labogena, France), using a capillary sequencer (ABI PRISM 3100 Genetic Analyzer; Applied Biosystems). Genotypes are available upon request.

### Statistical analysis

Administrative provinces were considered as sampling units for performing preliminary estimations of genetic polymorphism. The presence of null alleles was tested using FreeNA [22]: loci with estimated frequencies of null alleles *r* ≥ 0.2 could be considered to be potentially problematic for calculations. The allele frequencies, number of alleles, observed (*H*_*o*_), non-biased expected (*H*_*e*_) heterozygosity, and F-Statistics [23] were estimated using GENETIX *4.05.2*[24]. GENEPOP *4.07*[25] was used to evaluate departure from Hardy-Weinberg equilibrium and pairwise genic differentiation among breeds [26]. Allelic richness (*Ar*) was computed with the rarefaction method using FSTAT *2.9.3.2*[27]. Test significance was corrected with sequential Bonferroni correction on loci.

The matrix of Reynolds distances (*D*_*R*_; [28]) was computed using PHYLIP 3.69 [29]. Regarding the *D*_*R*_ distance, a NeighborNet [30] network was drawn using SPLITSTREE *4.8*[31].

Clustering approaches were performed, in a first step, on the 27 populations using a Bayesian clustering procedure implemented in STRUCTURE [32], considering a number of K clusters ranging from 1 to 16. For each K, 100 runs were performed with 100 000 iterations following a burn-in period of 100 000, under admixture and correlated allele frequency model. As consistency across runs seems to be an informative method for assessing species structure across breeds [33,34], we used CLUMPP [35] to estimate the similarity function G’ over runs for the different values of K, using LARGEKGREEDY algorithm. We selected a subset of runs that included the run with the highest number of similar runs (symmetric similarity coefficients SSC higher than 0.95) and the corresponding runs. We used this subset to compute a mean Q-matrix. In a second step, analysis was reduced to the 326 individuals with exact coordinates from Benin, Côte d’Ivoire, and Ghana. A clustering approach was also conducted under the same conditions for K = 1 to 6. On the basis of membership coefficients for K = 2, the genetic structure of the set was interpolated spatially with the Kriging approach, using the R procedure described by François [36]. Membership values, according to agroecological zones, were tested using *t*-test procedures.

Potential genetic introgression from commercial lines to local populations was investigated by computing the individual allele shared distance (*DAS*) [37] matrix for each country, including all individuals from commercial lines. Matrices and dendrograms were obtained using POPULATION *1.2.28*[38] and TREEPLOT *0.7*[39] respectively.

## Results

### Genetic variations

Amongst the 22 markers, 188 alleles were identified, with the number of alleles per marker ranging from 2 to 23 ( Additional file 1). When grouped by country or commercial lines altogether, total number of alleles ranged from 117 (commercial lines) to 156 (Côte d’Ivoire), number of private alleles ranged from 2 (Cameroon and Morocco) to 7 (Côte d’Ivoire), while allelic richness ranged from 4.85 (commercial lines) to 5.96 (Côte d’Ivoire).

*He* values ranged from 0.297 (WEL-A) to 0.665 (MAR-Mek) with a mean value of 0.560 (±0.078), according to the genetic diversity indices of the studied populations (Table 2). *Ar* (computed for populations with more than 10 individuals genotyped for each locus) values rose from 2.04 (WEL-A) to 4.84 (CIV-SCo) with a mean value of 3.93 (±0.73). *Fis* ranged from −0.081 (BEN-Oué) to 0.131 (MAR-Mek). After sequential Bonferroni correction, 6 populations showed a significant deficit of heterozygotes for 1 or 2 loci, and one population exhibited 1 locus with heterozygote excess. Only two locus x population combinations out of 616 were identified with a potentially null allele (*r* > 0.2; data not shown): MCW037 x CAM-Est and MCW330 x BEL-C. However, excluding these two loci had very minor effects on *Fis* and *He* (Wilcoxon test; *P-*value > 0.05), suggesting that null alleles are not the main cause of significant *Fis* values. Hence we chose to conserve all 22 loci. Testing population differentiation, 112 pairs of populations were found as non-significantly differentiated out of the 378 tests performed ( Additional file 2). All pairwise comparisons involving either commercial lines or the Moroccan population were significant. Within the 6 pairwise comparisons among Cameroon chicken populations, only 2 were significant. The CAM-Est sample could not be differentiated from several populations of Côte d’Ivoire (CIV-SCo and CIV-Lag) or Ghana (GHA-Ash, GHA-Eas and GHA-Wes). Furthermore, CIV-SCo was not differentiated from CAM-Cen and CAM-Sud and CIV-Lag was not differentiated from CAM-Cen. All the other non significant pairwise tests (100) involved pairwise comparisons within or among Benin, Côte d’Ivoire, and Ghana.

**Table 2:** Summary of genetic diversity measures across African and commercial populations

**sample**	***He***	***Ho***	***MNA***	**Ar (N > 10)**	***Fis***	***LEHWE***	***LDHWE***
BEN-Atl	0.534	0.512	4.64	4.06	0.041		
BEN-Lit	0.588	0.613	4.41	-	−0.044		
BEN-Cou	0.574	0.606	3.82	-	−0.060		
BEN-Mon	0.562	0.558	3.95	-	0.008		
BEN-Oué	0.550	0.591	3.55	-	−0.081		
BEN-Bor	0.548	0.540	5.14	3.95	0.013		
BEN-Don	0.526	0.503	4.14	3.81	0.046		
CIV-Agn	0.605	0.595	4.82	4.40	0.018		
CIV-SCo	0.638	0.616	5.32	4.84	0.036		1
CIV-Lag	0.612	0.574	4.77	4.40	0.064		
CIV-Lac	0.591	0.586	6.00	4.54	0.009		
CIV-NCo	0.553	0.528	5.05	4.30	0.045		1
GHA-Ash	0.565	0.561	3.64	-	0.009		
GHA-Eas	0.619	0.625	5.09	4.33	−0.010		
GHA-Wes	0.612	0.583	5.14	4.32	0.048		1
GHA-Nor	0.571	0.551	4.95	4.25	0.035		
GHA-UEa	0.510	0.503	3.77	3.58	0.014		
GHA-UWe	0.561	0.553	4.18	4.18	0.015	1	
MAR-Mek	0.665	0.579	5.36	4.48	0.131*		2
CAM-Cen	0.635	0.637	5.46	4.57	−0.003		
CAM-Est	0.634	0.664	3.36	-	−0.052		
CAM-Sud	0.632	0.647	5.55	4.54	−0.024		
CAM-ONO	0.632	0.613	4.73	4.21	0.032		1
BS-C	0.499	0.512	3.09	2.92	−0.025		
BS-D	0.481	0.475	3.55	3.13	0.012		
BD-B	0.482	0.484	3.14	2.92	−0.003		
WEL-A	0.297	0.295	2.14	2.04	0.008		
BEL-C	0.406	0.360	2.95	2.77	0.115*		2

*He*: non-biased expected heterozygosity; *Ho*: observed heterozygosity; *MNA*: mean number of alleles per locus; *Ar* (N > 10): allelic richness computed for populations with more than 10 individuals genotyped for each locus; Fis*: significant value after sequential bonferroni correction; *LD* number of Loci in heterozygote Deficiency/Excess, after sequential Bonferroni correction.

### Population relationships and clustering

**Figure 2:**
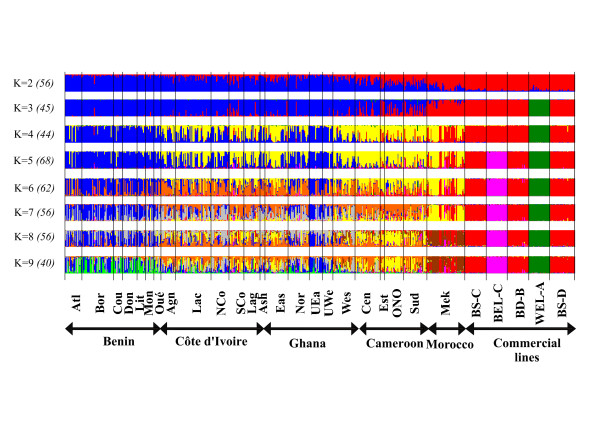
**Estimated membership coefficients of each individual to the inferred K cluster, with K = 2–9.** In brackets, the number of runs with similar solutions (SSC > 0.95) that has been used to compute the mean Q-matrix.

The use of the Bayesian clustering approach allows estimating the genetic structure within the population studied, using either the Q-matrix averaged over the most similar runs for K = 2 to 9 (Figure 2, see material and methods) or overall runs for K = 2 to 16 ( Additional file 3). As K increased, the likelihood (Ln(P(D))) increased until K = 9 and stabilized afterwards, while its standard deviation increased ( Additional file 4), indicating that K = 9 captures the major structure proportion present in the data, and that only a minor proportion of the genetic structure is described by higher K values [40,41]. From K = 2, most African chicken populations appeared clearly differentiated from commercial lines and the Moroccan population, with Cameroon chicken populations showing intermediate results (Figure 2). As K increased, the two layer lines appeared to be quickly differentiated (for K = 3 and 5), while some differentiation appeared within the African populations. All individuals from Benin and the Ghanaian chickens from GHA-UEa and GHA-UWe regions formed one cluster, which differed from the cluster formed by the remaining Ghanaian individuals, Côte d’Ivoire, Cameroonian, and most of Moroccan samples. However, these results could not be generalized for African chicken populations at the individual level, and there was a relatively high heterogeneity of membership coefficients within populations, particularly in comparison with commercial lines. The genetic structure appeared more complex as K increased. Regarding Morocco for instance, the population could be divided into two groups according to their admixture coefficient: the first one, more numerous, was found close to African populations but was completely differentiated from K = 8, while the second one included individuals belonging to the same cluster as broiler lines (14 individuals with membership coefficients greater than 0.5 for K = 6). These individuals were differentiated from broiler samples only from K = 14 ( Additional file 3). It was also observed that a few individuals from Morocco shared a relatively high membership coefficient for the cluster specific to the brown-egg commercial line (BEL-C): for K = 6, 3 individuals had membership coefficients between 0.42 and 0.46. When using the Q-matrix averaged over the 100 runs ( Additional file 3), we noticed that the five commercial lines could be clearly differentiated. The white-egg layer (WEL-A) was, in general, the first one to differentiate within this group, which was expected since it derives from the single breed White Leghorn. The other lines shared Asiatic origins; however, different breed histories led to their differentiation as K increased.

**Figure 3:**
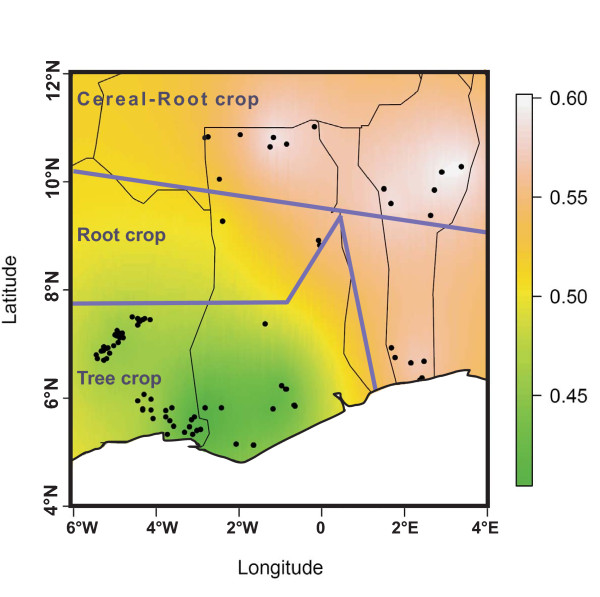
**Geographical interpolation of structure result (K = 2) on individual probabilities to belong to cluster 1 (***q*** membreships from 0.4 (green) to 0.6 (white), for local chicken samples from Benin, Côte d’Ivoire and Ghana (latitude and longitude in degrees).** Major Farming Systems (MFS) are indicated according to Dixon *et al.*[42]. The purple lines delineate the MFS.

Structure analysis was then restricted to individuals sampled in the area including Ghana, Benin, and Côte d’Ivoire. Following the higher likelihood found for K = 2 ( Additional file 5), the results interpolated using geographical coordinates are given in Figure 3, showing that individuals were distributed along a north-east/south-west cline. The north-east side included all Beninese samples, GHA-UEa, GHA-UWe, and GHA-Nor (the results being intermediate for one of the GHA-Nor sampling sites). The largest membership values were found within BEN-Don and GHA-UEa (cluster 1 membership >0.62) regions. All the samples from Côte d’Ivoire, GHA-Ash, GHA-Eas, and GHA-Wes were found at the south-west side. The lowest values were found within CIV-SCo and GHA-Eas (cluster 1 membership <0.40).

**Figure 4:**
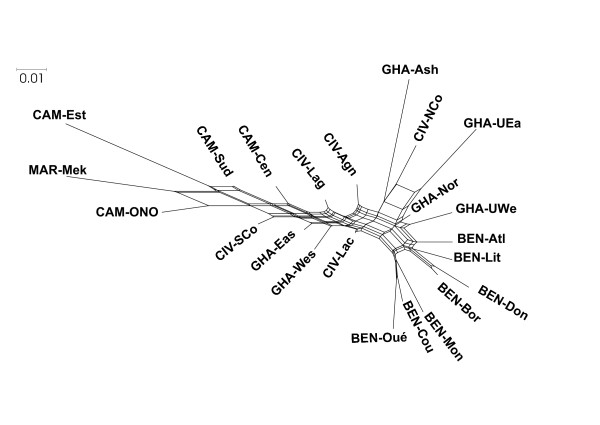
**Neighbour-Net for the 23 African local chicken populations, based on Reynolds* D***_***R ***_**distance.**

According to the *D*_*R*_ distances ( Additional file 2), the Neighbor-Net network restricted on African populations (Figure 4) confirmed the results found through geographical interpolation with regards to the genetic relationships among populations from Côte d’Ivoire, Ghana, and Benin. According to the network, chicken samples from Cameroon and Morocco were found to be genetically closer to the south-west group; this was in agreement with STRUCTURE general results (Figure 2). Adding commercial lines did not change the results ( Additional file 6). Those lines were found to be largely differentiated in comparison to the other populations.

Individual dendrograms (Additional files 7, 8, 9, 10 and 11) established for the different countries showed that some local chickens were intermingled with chickens from commercial lines. If no individuals from Benin were classified with commercial chickens, this was the case for a few individuals from Côte d’Ivoire and Ghana (1 and 3 respectively), all of them being found at the periphery of commercial clusters. A much larger number of individuals from Cameroon were misclassified, while chickens from Morocco were separated into different clusters distributed across commercial lines. When compared with individual membership results from the STRUCTURE approach (for K = 6), chickens with *qi* > 0.2 for clusters specific to commercial lines were in general clustered in small groups (Additional files 10 and 11), even if they were not always assigned close to commercial clusters. Note also that the BEL cluster, if always clearly defined, plotted with the other selected in three cases (Benin, Ghana, and Morocco, Additional files 7, 9 and 11 respectively) but was positioned within the local population for the two other cases (Côte d’Ivoire and Cameroon, Additional files 8 and 10 respectively).

## Discussion

### Gene diversity and gene flow

Several studies have been published which include a large collection of chicken populations genotyped with microsatellite markers [8,10,20,21,43,44]. Heterozygosity levels reported here, for the 23 local African chicken populations, were similar to the values found in other scavenging populations, in Africa or in Asia, ranging from 0.53 to 0.7 [8,9,11,15,16,44-46] and being larger than those found in standardized (fancy) breeds (0.28–0.63) or commercial lines (0.34–0.63) [10,21,47]. Such levels can be expected for domestic populations which are not selected and have not been submitted to any bottlenecks in a recent past.

Since introgression from commercial lines may constitute a major issue for conservation and management of local chicken populations [6,11], we tried to assess the evidence and impact of such gene flows. Two contrasted situations were observed. In the first case, involving populations from Benin, Ghana, and Côte d’Ivoire, introgression can be considered as negligible or limited, although introduction of commercial lines in these countries has taken place (see [48,49] for some examples in Côte d’Ivoire). In the second case, observed in Cameroon and Morocco, clustering results and individuals’ dendrograms suggest that such gene flows have impacted the population structure to some extent. Observations on phenotypic variability may be used to confirm this suggestion because some traits are quite typical of commercial lines. This is the case of the yellow shank phenotype due to a recessive autosomal mutation at the *W* locus [50] found in all commercial layers, either white-egg or brown-egg, and in several broiler lines, whereas the wild type is a grey-blue shank colour. Previous surveys showed that the yellow shank phenotype is quite rare in Benin (5% [14]) and Côte d’Ivoire (15% [51]) or in other African countries such as Senegal (4% [52]), while it is much more common either in Cameroon (31 to 38% [53,54]) or Morocco (60% in the studied sample; Benabdeljelil, personal observation). Furthermore, the presence of the *W* mutation was found to be correlated with an improvement of anatomical traits in Cameroon [55]. In Cameroon also, the dwarf phenotype (allele *DW*) found in low frequency in some regions, may also indicate commercial introgression from broiler lines [54].

As in other places in Africa, several cooperating programs (FAO, bilateral cooperation, NGO…) were set up in these countries in order to improve poultry production, some of these involving the introduction of exotic cockerels for genetic improvement. Differences in the temporal framework of these programs could be, at least partially, responsible for the contrasting patterns of genetic introgression observed in our study. On the one hand, long-term operations took place in Morocco and Cameroon. In Morocco [56], the first poultry station was built in 1920 at Meknes. More recently, commercial hatcheries could supply traditional farmers with either male chicks of commercial layer lines or crossbred chicks obtained from Rhode Island and slow-growing meat type lines, such as French label and Barred Plymouth Rock. In Cameroon [57], supply of chicks to farmers was conducted by three pilot stations from 1960 to 1984. In these cases, introduction of exotic chickens within the local livestock was more or less continuous for a long time. On the other hand, Ghana [58], Benin [59], or Côte d’Ivoire [49] operations were characterized by short-term actions: some programs for poultry development, with the introduction of limited numbers of cockerels, have been registered and, according to our results, had a very limited genetic impact on the local chicken gene pool.

Yet, taking into account the duration of exotic cocks introduction, genetic introgression appears finally relatively low even in Morocco and Cameroon. Limited impact of introductions may be explained by a poor adaptation of the genotypes introduced to local conditions or by the consumer’s preference. Indeed, commercial lines have been counter-selected against broodiness, and then females have lost the ability to incubate their eggs naturally, whereas this ability is particularly important for self propagation of village chickens in harsh environments. Although the genetic determinism of broodiness is still under study [60], it is expected that F_1_ females from exotic cocks are not as successful as the local hens in natural reproduction and chick rearing. Furthermore, commercial chicks have been selected with an optimum feeding system, and may not satisfy their nutritional needs in scavenging conditions. According to some authors [3,61], there is a consumer preference for local chickens, both for meat and eggs. Such preference has the effect that the sale price of local chickens (and eggs) is higher than that of products from commercial lines in African countries, including those studied here [49,56-59,62,63]. We also observed that phenotypic variability is very important due to the diversified social uses of local chickens [51,54,64] such as religious rituals or indigenous pharmacopeia. Some traits may be retained from industrial birds when they have a relative advantage, particularly the white color, as described by [6] who observed that a white plumage color was a peace symbol in Benin. Plumage color is determined by major genes which may interact with each other. Some of these genes may have been introgressed from commercial lines into local chickens, as in the case of the yellow shank due to an autosomal recessive mutation [50].

### Chicken population structure

Usually, scavenging chicken populations do not exhibit a typical breed structure [11,15,45,65] even if some more or less marked differentiation could eventually be found [9,46], in particular for large geographical data sets involving several African countries [16]. When considering our data globally, we found the same result. Yet, some relations with climatic conditions may be observed: local chickens from regions with high precipitations (>1400 mm/year [66]), i.e. south-eastern Côte d’Ivoire, southern Ghana, and Cameroon, seemed to share a similar genetic background according to Structure results (Figure 2) in contrast to chickens of the other regions with lower precipitations. The fact that a similar diversity pattern was found both in Cameroon and in southern Ghana/Côte d’Ivoire seems to indicate that such an observation could not result from genetic drift only. Restricting the analysis to Côte d’Ivoire, Ghana, and Benin, made it possible to identify a north-east/south-west cline illustrated in Figure 3. According to the FAO [42], this area may be divided into three Major Farming Systems (MFS). Considering our genetic differentiation results (Figure 3), the Tree Crop Farming System (TCFS) corresponds to the south-west side of the cline, involving Côte d’Ivoire and southern Ghana, while the Cereal-Root Crop Farming System (C-RCFS) lies on the north-east side (northern part of Ghana and Benin). An intermediate area corresponding to the Root Crop Farming System (RCFS) and including middle Ghana and southern Benin exhibited a more similar pattern to C-RCFS, both for genetic data and farming system. On the basis of Structure results, the three zones showed significantly different membership values (*P* < 0.01 for RCFS/C-RCFS comparison and *P* < 0.001 for the two other ones). Within the RCFS, according to surveys conducted in Benin [14], the weight and body measures of Savannah chickens (north of Benin) were found to be significantly higher than those of Forest chickens (south of Benin). Experimental comparisons [67] showed the same difference, suggesting that it could correspond, to some extent, to genetic effects, although no significant difference (*P* = 0.059) was observed between animals of northern and southern part of Benin, according to the Structure analysis restricted to Benin, Ghana, and Côte d’Ivoire (based on the individuals *q*_*i*_). The number of markers used may be too limited to ensure detection of a genetic differentiation on a quantitative trait such as growth.

Human settlements and migrations may constitute another driving factor for the genetic structure of chicken populations. Although not well documented, there is a lot of movement of chickens between these countries as a result of long history of trade and migration. In particular, a large part of the human groups living in the south-east part of Côte d’Ivoire and south-west part of Ghana belong to the same ethnic group, namely the Akan, originating from Ghana [68,69]. Among others, these groups share most of the same cultural practices [70] implying similar uses of chicken resources [51] as part of this cultural background. Nevertheless, the admixture pattern could be related to commercial exchanges amplified by the high mobility of the people and the fact that chickens can be easily freighted [51], limiting the extent of genetic differentiation among areas.

These agroecological, cultural and demographic factors could result in genetic drift, gene flows or adaptation phenomena, and therefore explain the genetic structure pattern found across these countries. On the basis of the present data, it is, however, difficult to assess to what extent each of these factors has impacted genetic differentiation of these chicken populations.

## Conclusion

African local chickens form a highly variable gene pool which constitutes a valuable resource for human populations. There is a large number of driving forces playing either for or against population differentiation of local chickens. Molecular studies bring complementary information to social surveys and phenotypic data, and allows to set up an integrated program of characterization and conservation of indigenous populations, as recommended by the FAO [1,71]. In this study, we were able to prove that such differentiation may exist among chicken populations, nevertheless, the relative importance of climatic influences and social practices are difficult to disentangle. Further analysis with a higher density of markers is necessary to ascertain the genetic structure of local chicken populations with a higher accuracy, and landscape genomics approaches would be useful to connect genetic differentiation with environmental conditions [72].

## Competing interest

The authors declare that they have no competing interests.

## Authors’ contributions

XR, IAKY, BBK, CVYG, KB and MTB conceived the project; IAKY, BBK, CVYG, ROA, NEL, JCF, KB, XR and BB collected the samples and data; XR and GL analysed the data; XR, GL and MTB led the writing, and all the authors participated in the discussion. All authors read and approved the final manuscript.

## Supplementary Material

Additional file 1 Summary of polymorphic measures for microsatellite markers. For each and over all populations or within each African country’s population or commercial line, the following information are given: allele range, number of alleles (*A*), number of private alleles (*Ap*) and allelic richness (*Ar*). Click here for file

Additional file 2 Pairwise genetic distances (*D*_*R*_) and levels of significance of genic differentiation among the 28 chicken populations. *D*_*R*_ values are above the diagonal and levels of significance are below the diagonal. For population codes, see Table 1. Click here for file

Additional file 3 STRUCTURE analysis involving all 28 populations (23 African local chicken populations and 5 commercial lines), for K = 2-16, using Q-matrix averaged overall 100 runs. Click here for file

Additional file 4 STRUCTURE analysis involving all 28 populations (23 African local chicken populations and 5 commercial lines). Evolution of (a) likelihood *Ln(P(D))* and (b) similarity function *G’* according to the number of cluster K (K = 1 to 16). Click here for file

Additional file 5 STRUCTURE analysis restricted to the 18 African local chicken populations from Ghana, Benin and Côte d’Ivoire. Evolution of likelihood *Ln(P(D))* according to the number of cluster K (K = 1 to 6). Click here for file

Additional file 6 Neighbor-Net for the complete dataset (23 African local chicken populations and 5 commercial lines), based on Reynolds D_*R*_ distance. Click here for file

Additional file 7 Individual neighbor-joining dendrogram based on *DAS* distance among 242 samples representing local chickens from Benin (n = 113) and 5 commercial lines (n = 129). red: Benin; orange: BS-D; dark green: BS-C; dark blue: Bel-C; light green: Wel-A; light blue: BD-B; black star indicates individuals with *q*_*i*_ > 0.2, for clusters specific to commercial lines (STRUCTURE analysis for K = 6). Click here for file

Additional file 8 Individual neighbor-joining dendrogram based on *DAS* distance among 250 samples representing local chickens from Côte d’Ivoire (n = 121) and 5 commercial lines (n = 129). red: Côte d’Ivoire; orange: BS-D; dark green: BS-C; dark blue: Bel-C; light green: Wel-A; light blue: BD-B; black star indicates individuals with *q*_*i*_ > 0.2, for clusters specific to commercial lines (STRUCTURE analysis for K = 6). Click here for file

Additional file 9 Individual neighbor-joining dendrogram based on *DAS* distance among 241 samples representing local chickens from Ghana (n = 112) and 5 commercial lines (n = 129). red: Ghana; orange: BS-D; dark green: BS-C; dark blue: Bel-C; light green: Wel-A; light blue: BD-B; black star indicates individuals with *q*_*i*_ > 0.2, for clusters specific to commercial lines (STRUCTURE analysis for K = 6). Click here for file

Additional file 10 Individual neighbor-joining dendrogram based on *DAS* distance among 214 samples representing local chickens from Cameroon (n = 85) and 5 commercial lines (n = 129). red: Cameroon; orange: BS-D; dark green: BS-C; dark blue: Bel-C; light green: Wel-A; light blue: BD-B; black star indicates individuals with *q*_*i*_ > 0.2, for clusters specific to commercial lines (STRUCTURE analysis for K = 6). Click here for file

Additional file 11 Individual neighbor-joining dendrogram based on *DAS* distance among 175 samples representing local chickens from Morocco (n = 46) and 5 commercial lines (n = 129). red: Morocco; orange: BS-D; dark green: BS-C; dark blue: Bel-C; light green: Wel-A; light blue: BD-B; black star indicates individuals with *q*_*i*_ > 0.2, for clusters specific to commercial lines (STRUCTURE analysis for K = 6). Click here for file
