# Supplementary material for: Gene diversity, agroecological structure and introgression patterns among village chicken populations across North, West and Central Africa
Source: BMC Genet. 2012 May 7;13:34. doi: 10.1186/1471-2156-13-34 (PMC3411438; doi:10.1186/1471-2156-13-34)
Supplement: Additional file 1 — Summary of polymorphic measures for microsatellite markers. For each and over all populations or within each African country’s population or commercial line, the following information are given: allele range, number of alleles (A), number of private alleles (Ap) and allelic richness (Ar). [file 1471-2156-13-34-S1.pdf]

**Additional file 1 – Summary of polymorphic measures for microsatellite markers.**

For each and over all populations or within each African country's population or commercial line, the following information are given: allele range, number of alleles (*A*), number of private alleles (*Ap*) and allelic richness (*Ar*).

| | Over the 28 populations | | Number of alleles for each African country or commercial lines | | | | | |
|---|---|---|---|---|---|---|---|---|
| Locus | Allele range | *A* | Benin (N=113) | Côte d'Ivoire (N=117) | Ghana (N=112) | Cameroon (N=85) | Morocco (N=45) | Commercial lines (N=129) |
| | | | *A* (*Ap*) | *A* (*Ap*) | *A* (*Ap*) | *A* (*Ap*) | *A* (*Ap*) | *A* (*Ap*) |
| ADL268 | 101-121 | 7 | 5 | 5 | 5 | 5 | 5 | 7 (2) |
| ADL278 | 110-124 | 10 | 6 | 9 (2) | 7 (1) | 7 | 7 | 5 |
| ADL112 | 119-129 | 6 | 5 | 6 | 5 | 5 | 4 | 6 |
| MCW295 | 83-103 | 10 | 10 (1) | 9 | 9 | 8 | 5 | 7 |
| MCW216 | 134-149 | 9 | 8 | 7 | 7 | 7 | 5 | 6 |
| MCW014 | 162-180 | 7 | 4 | 6 | 4 | 6 | 3 | 5 |
| MCW098 | 255-257 | 2 | 2 | 2 | 2 | 2 | 2 | 2 |
| LEI234 | 212-371 | 23 | 15 | 20 (2) | 17 (1) | 15 | 13 (1) | 10 (1) |
| MCW111 | 97-111 | 7 | 7 (1) | 6 | 6 | 5 | 4 | 3 |
| MCW078 | 134-142 | 5 | 4 | 5 | 5 | 5 | 4 | 4 |
| MCW222 | 216-222 | 4 | 4 | 4 | 4 | 4 | 4 | 4 |
| MCW183 | 292-326 | 11 | 8 | 10 | 11 (1) | 7 | 8 | 6 |
| LEI094 | 246-285 | 16 | 9 | 11 (2) | 7 | 11 | 8 (1) | 8 (1) |
| MCW069 | 154-174 | 9 | 6 | 9 | 7 | 8 | 6 | 4 |
| MCW034 | 214-244 | 14 | 11 | 10 | 11 | 11 | 9 | 9 |
| MCW037 | 150-156 | 6 | 6 | 6 | 6 | 6 | 6 | 6 |

| | | | | | | | | |
|---|---|---|---|---|---|---|---|---|
| MCW067 | 172-182 | 6 | 5 | 3 | 5 | 6 (1) | 3 | 4 |
| MCW206 | 218-247 | 10 | 7 | 8 | 7 | 7 (1) | 5 | 6 |
| MCW081 | 109-131 | 9 | 7 (1) | 7 (1) | 7 (1) | 5 | 6 | 4 |
| MCW248 | 213-221 | 4 | 3 | 4 | 4 | 4 | 3 | 3 |
| LEI166 | 251-261 | 4 | 4 | 4 | 3 | 3 | 3 | 3 |
| MCW330 | 254-286 | 9 | 6 (1) | 5 | 7 | 7 | 5 | 5 |
| global | | 188 | 142 (4) | 156 (7) | 146 (4) | 144 (2) | 118 (2) | 117 (4) |
| *Ar* | | | 5.51 | 5.96 | 5.58 | 5.85 | 5.32 | 4.85 |
