# Supplementary material for: Gene diversity, agroecological structure and introgression patterns among village chicken populations across North, West and Central Africa
Source: BMC Genet. 2012 May 7;13:34. doi: 10.1186/1471-2156-13-34 (PMC3411438; doi:10.1186/1471-2156-13-34)
Supplement: Additional file 2 — Pairwise genetic distances (DR) and levels of significance of genic differentiation among the 28 chicken populations. DR values are above the diagonal and levels of significance are below the diagonal. For population codes, see Table 1. [file 1471-2156-13-34-S2.pdf]

Additional file 2 – Pairwise genetic distances (D_R) and levels of significance of genic differentiation among the 28 chicken populations. D_R values are above the diagonal and levels of significance are below the diagonal. For population codes, see Table 1.

	BEN-Atl	BEN-Bor	BEN-Cou	BEN-Don	BEN-Lit	BEN-Mon	BEN-Oué	CIV-Agn	CIV-Lac	CIV-NCo	CIV-Sco	CIV-Lag	GHA-Ash	GHA-Eas
BEN-Atl		0.027	0.038	0.036	0.031	0.046	0.050	0.043	0.035	0.057	0.065	0.047	0.064	0.054
BEN-Bor	NS		0.037	0.019	0.029	0.040	0.040	0.052	0.036	0.059	0.064	0.050	0.071	0.049
BEN-Cou	NS	NS		0.047	0.032	0.036	0.027	0.056	0.040	0.070	0.068	0.053	0.085	0.053
BEN-Don	NS	NS	NS		0.034	0.059	0.054	0.053	0.048	0.065	0.081	0.060	0.080	0.055
BEN-Lit	NS	NS	NS	NS		0.037	0.052	0.051	0.046	0.079	0.066	0.049	0.088	0.054
BEN-Mon	NS	NS	NS	NS	NS		0.041	0.059	0.045	0.079	0.071	0.049	0.091	0.062
BEN-Oué	NS	NS	NS	NS	NS	NS		0.066	0.041	0.069	0.081	0.060	0.085	0.072
CIV-Agn	NS	***	NS	*	NS	NS	NS		0.026	0.038	0.044	0.024	0.071	0.030
CIV-Lac	*	***	*	***	*	NS	NS	NS		0.032	0.035	0.023	0.061	0.023
CIV-NCo	**	***	***	*	**	**	NS	NS	NS		0.068	0.048	0.068	0.045
CIV-Sco	***	***	NS	***	NS	NS	NS	NS	NS	***		0.033	0.080	0.033
CIV-Lag	*	***	NS	**	NS	NS	NS	NS	NS	NS	NS		0.082	0.026
GHA-Ash	NS	NS	NS	NS	NS	NS	NS	NS	NS	NS	NS	NS		0.070
GHA-Eas	***	***	**	***	*	***	***	NS	**	**	NS	NS	NS	
GHA-Nor	*	***	NS	**	NS	NS	NS	NS	NS	NS	**	NS	NS	NS
GHA-UEa	NS	***	***	NS	*	*	NS	NS	**	NS	***	**	NS	***
GHA-UWe	NS	**	NS	NS	NS	NS	NS	NS	NS	NS	*	NS	NS	**
GHA-Wes	***	***	NS	***	NS	NS	NS	NS	***	**	NS	NS	NS	**
CAM-Cen	***	***	***	***	***	***	**	***	***	***	NS	NS	**	***
CAM-Est	***	***	***	***	*	***	***	**	**	***	NS	NS	NS	NS
CAM-ONO	***	***	***	***	***	***	***	***	***	***	*	***	***	***
CAM-Sud	***	***	***	***	***	***	***	***	***	***	NS	*	**	***
MAR-MekT	***	***	***	***	***	***	***	***	***	***	***	***	***	***
BS-C	***	***	***	***	***	***	***	***	***	***	***	***	***	***
BEL-C	***	***	***	***	***	***	***	***	***	***	***	***	***	***
BD-B	***	***	***	***	***	***	***	***	***	***	***	***	***	***
WEL-A	***	***	***	***	***	***	***	***	***	***	***	***	***	***
BS-D	***	***	***	***	***	***	***	***	***	***	***	***	***	***

Additional file 2 (end)

	GHA-Nor	GHA-UEa	GHA-UWe	GHA-Wes	CAM-Cen	CAM-Est	CAM-ONO	CAM-Sud	MAR-Mek	BS-C	BEL-C	BD-B	WEL-A	BS-D
BEN-Atl	0.038	0.062	0.040	0.049	0.058	0.151	0.109	0.083	0.150	0.240	0.343	0.298	0.397	0.274
BEN-Bor	0.038	0.055	0.041	0.058	0.068	0.141	0.094	0.085	0.145	0.236	0.335	0.283	0.377	0.266
BEN-Cou	0.041	0.083	0.052	0.040	0.060	0.140	0.088	0.074	0.126	0.227	0.318	0.271	0.382	0.257
BEN-Don	0.046	0.062	0.043	0.071	0.077	0.161	0.110	0.098	0.164	0.256	0.358	0.301	0.396	0.274
BEN-Lit	0.043	0.077	0.043	0.053	0.060	0.130	0.090	0.075	0.129	0.218	0.327	0.278	0.372	0.255
BEN-Mon	0.042	0.079	0.060	0.053	0.070	0.136	0.098	0.083	0.137	0.244	0.306	0.277	0.392	0.265
BEN-Oué	0.042	0.064	0.053	0.057	0.075	0.158	0.109	0.086	0.151	0.250	0.337	0.287	0.416	0.282
CIV-Agn	0.025	0.053	0.037	0.035	0.040	0.119	0.078	0.054	0.103	0.184	0.293	0.250	0.377	0.215
CIV-Lac	0.017	0.047	0.033	0.032	0.038	0.105	0.063	0.039	0.095	0.183	0.280	0.236	0.379	0.215
CIV-NCo	0.039	0.052	0.057	0.047	0.070	0.145	0.104	0.077	0.130	0.207	0.320	0.263	0.414	0.223
CIV-Sco	0.052	0.090	0.076	0.045	0.039	0.071	0.053	0.040	0.074	0.146	0.266	0.237	0.345	0.176
CIV-Lag	0.035	0.063	0.045	0.030	0.027	0.096	0.062	0.037	0.094	0.166	0.282	0.231	0.368	0.199
GHA-Ash	0.060	0.080	0.084	0.071	0.089	0.154	0.136	0.104	0.160	0.238	0.364	0.302	0.416	0.265
GHA-Eas	0.032	0.078	0.051	0.033	0.030	0.092	0.054	0.035	0.078	0.166	0.256	0.218	0.330	0.192
GHA-Nor		0.046	0.026	0.038	0.054	0.129	0.082	0.058	0.114	0.203	0.300	0.255	0.389	0.240
GHA-UEa	NS		0.052	0.086	0.105	0.169	0.148	0.106	0.170	0.250	0.360	0.310	0.450	0.290
GHA-UWe	NS	NS		0.054	0.066	0.141	0.105	0.073	0.142	0.220	0.334	0.263	0.401	0.258
GHA-Wes	**	***	*		0.029	0.100	0.067	0.049	0.087	0.182	0.271	0.233	0.365	0.203
CAM-Cen	***	***	***	*		0.084	0.047	0.030	0.085	0.165	0.255	0.230	0.340	0.189
CAM-Est	***	***	***	NS	NS		0.104	0.059	0.107	0.144	0.291	0.259	0.344	0.187
CAM-ONO	***	***	***	***	***	NS		0.050	0.064	0.167	0.247	0.200	0.329	0.178
CAM-Sud	***	***	***	***	NS	NS	***		0.063	0.137	0.245	0.204	0.320	0.170
MAR-Mek	***	***	***	***	***	***	***	***		0.154	0.216	0.176	0.310	0.128
BS-C	***	***	***	***	***	***	***	***	***		0.394	0.277	0.438	0.217
BEL-C	***	***	***	***	***	***	***	***	***	***		0.342	0.536	0.375
BD-B	***	***	***	***	***	***	***	***	***	***	***		0.471	0.320
WEL-A	***	***	***	***	***	***	***	***	***	***	***	***		0.441
BS-D	***	***	***	***	***	***	***	***	***	***	***	***	***	

NS: not significant; *: $p<0.05$; **: $p<0.01$; ***: $p<0.001$
