# Supplementary material for: Gene diversity, agroecological structure and introgression patterns among village chicken populations across North, West and Central Africa
Source: BMC Genet. 2012 May 7;13:34. doi: 10.1186/1471-2156-13-34 (PMC3411438; doi:10.1186/1471-2156-13-34)
Supplement: Additional file 3 — STRUCTURE analysis involving all 28 populations (23 African local chicken populations and 5 commercial lines), for K = 2-16, using Q-matrix averaged overall 100 runs. [file 1471-2156-13-34-S3.pdf]

(a)

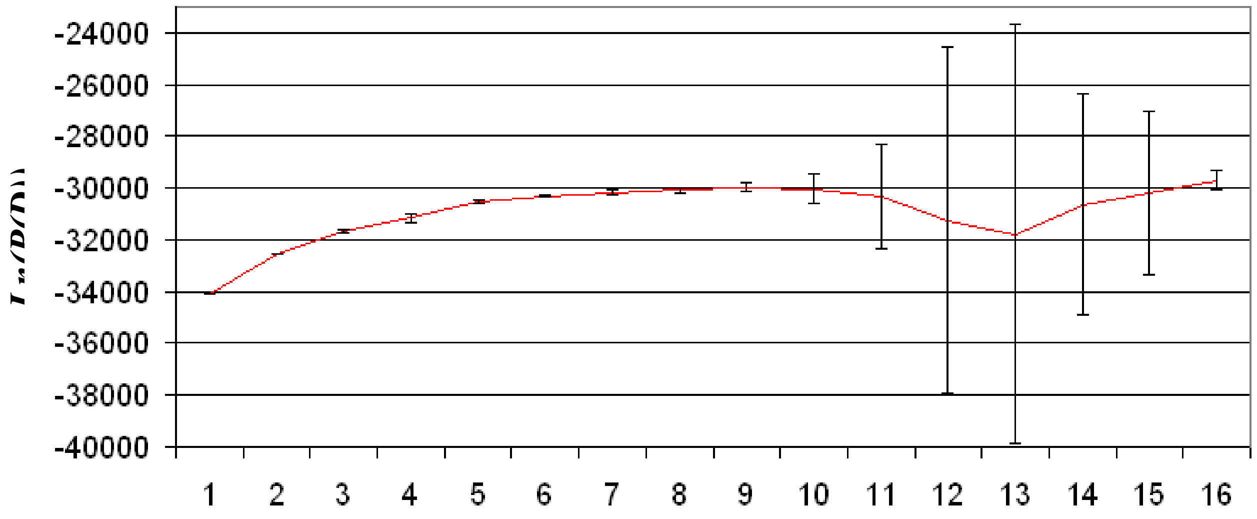

(b)

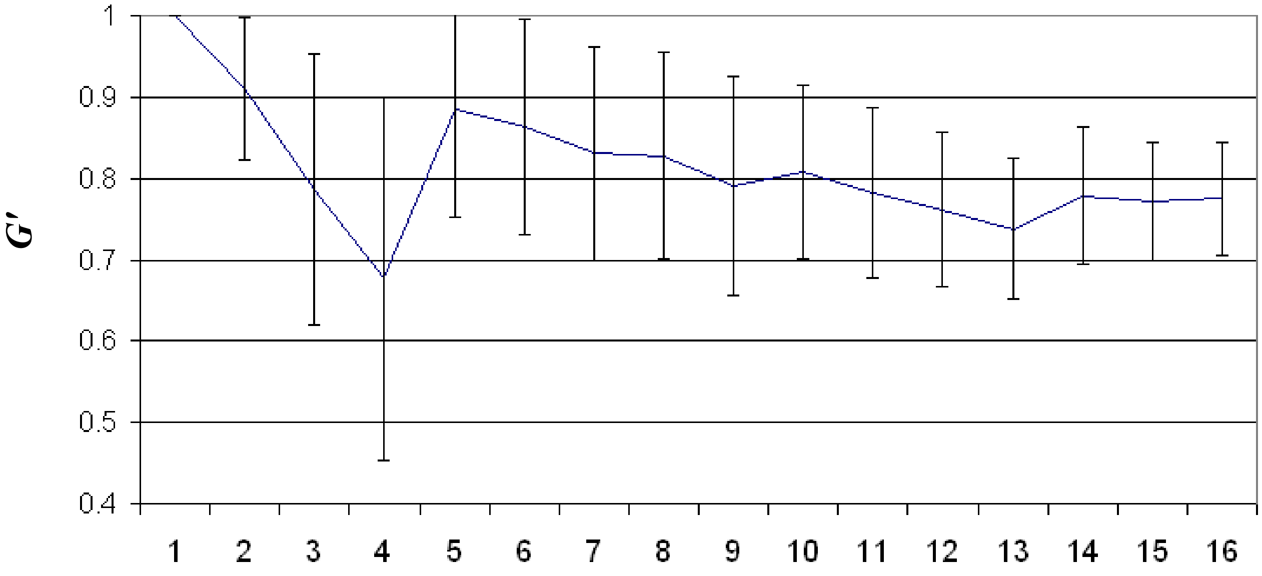

Additional file 3 – STRUCTURE analysis involving all 28 populations (23 African local chicken populations and 5 commercial lines).

Evolution of (a) likelihood *Ln(P(D))* and (b) similarity according to the number of cluster K (K=1 to 16).
