# Supplementary material for: Gene diversity, agroecological structure and introgression patterns among village chicken populations across North, West and Central Africa
Source: BMC Genet. 2012 May 7;13:34. doi: 10.1186/1471-2156-13-34 (PMC3411438; doi:10.1186/1471-2156-13-34)
Supplement: Additional file 4 — STRUCTURE analysis involving all 28 populations (23 African local chicken populations and 5 commercial lines). Evolution of (a) likelihood Ln(P(D)) and (b) similarity function G’ according to the number of cluster K (K = 1 to 16). [file 1471-2156-13-34-S4.pdf]

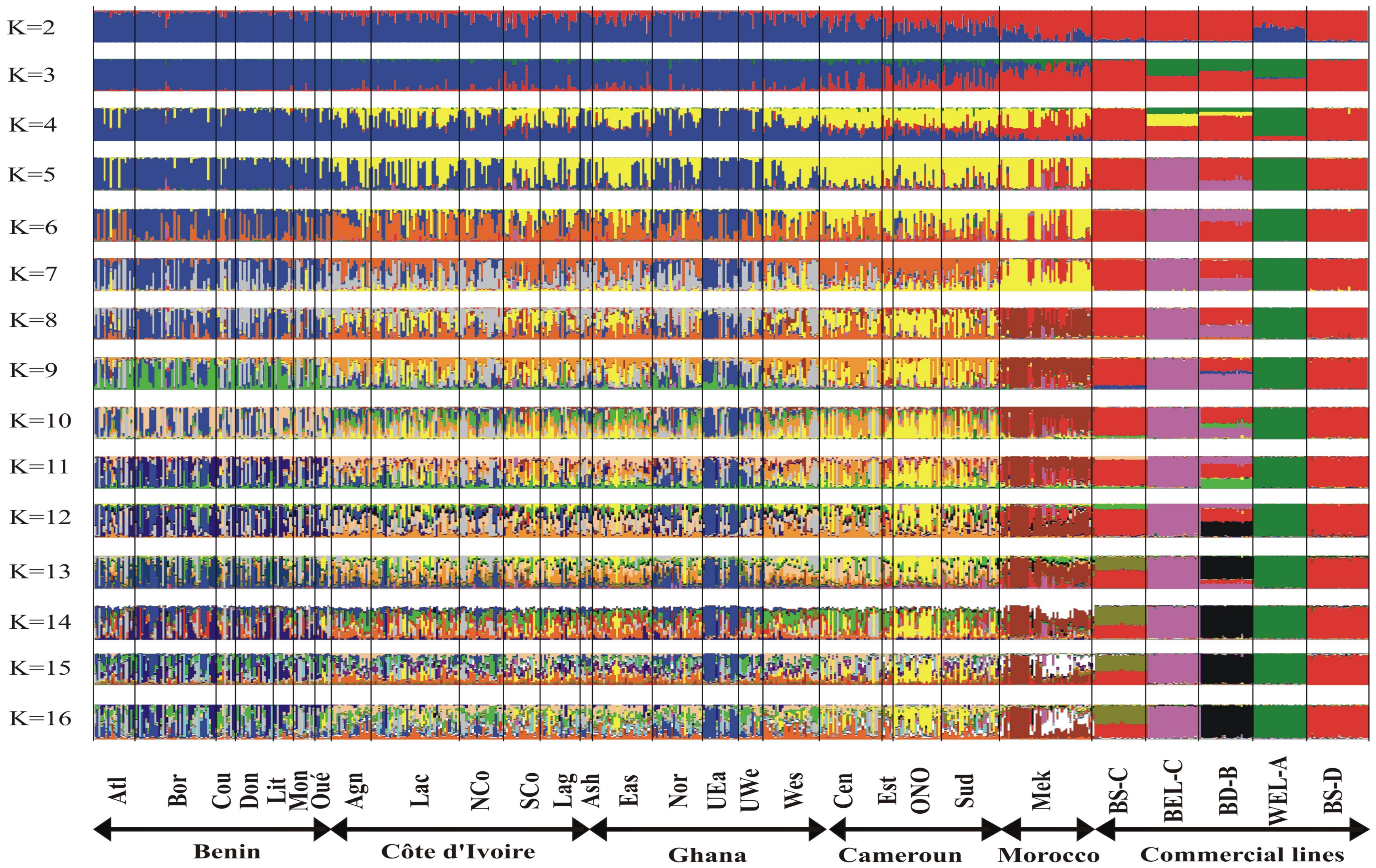

Additional file 4 – STRUCTURE analysis involving all 28 populations (23 African local chicken populations and 5 commercial lines), for K=2-16, using Q-matrix averaged overall 100 runs.
