# Supplementary material for: Gene diversity, agroecological structure and introgression patterns among village chicken populations across North, West and Central Africa
Source: BMC Genet. 2012 May 7;13:34. doi: 10.1186/1471-2156-13-34 (PMC3411438; doi:10.1186/1471-2156-13-34)
Supplement: Additional file 5 — STRUCTURE analysis restricted to the 18 African local chicken populations from Ghana, Benin and Côte d’Ivoire. Evolution of likelihood Ln(P(D)) according to the number of cluster K (K = 1 to 6). [file 1471-2156-13-34-S5.pdf]

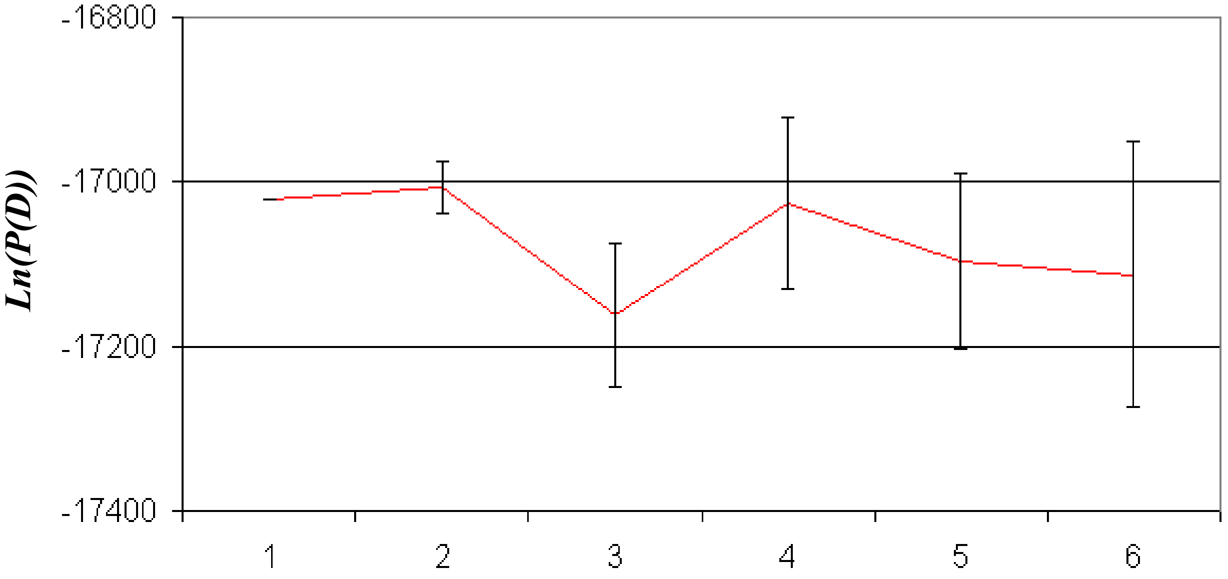

Additional file 5 – STRUCTURE analysis restricted to the 18 African local chicken populations from Ghana, Benin and Côte d'Ivoire.

Evolution of likelihood *Ln(P(D))* according to the number of cluster K (K=1 to 6).
