# Supplementary material for: Gene diversity, agroecological structure and introgression patterns among village chicken populations across North, West and Central Africa
Source: BMC Genet. 2012 May 7;13:34. doi: 10.1186/1471-2156-13-34 (PMC3411438; doi:10.1186/1471-2156-13-34)
Supplement: Additional file 6 — Neighbor-Net for the complete dataset (23 African local chicken populations and 5 commercial lines), based on Reynolds DR distance. [file 1471-2156-13-34-S6.pdf]

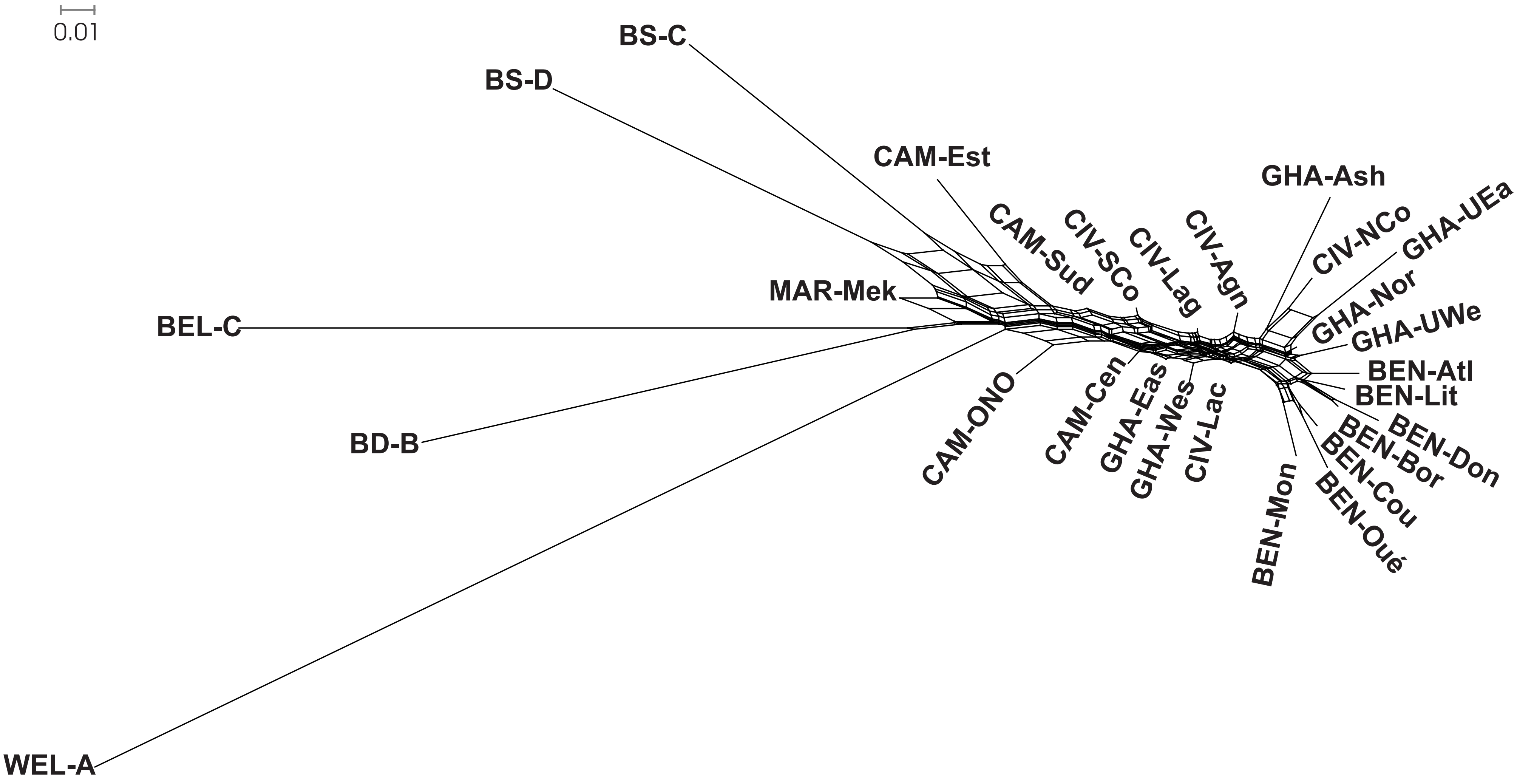

Additional file 6 – Neighbor-Net for the complete dataset (23 African local chicken populations and 5 commercial lines), based on Reynolds DR distance.
