# Supplementary material for: Gene diversity, agroecological structure and introgression patterns among village chicken populations across North, West and Central Africa
Source: BMC Genet. 2012 May 7;13:34. doi: 10.1186/1471-2156-13-34 (PMC3411438; doi:10.1186/1471-2156-13-34)
Supplement: Additional file 11 — Individual neighbor-joining dendrogram based on DAS distance among 175 samples representing local chickens from Morocco (n = 46) and 5 commercial lines (n = 129). red: Morocco; orange: BS-D; dark green: BS-C; dark blue: Bel-C; light green: Wel-A; light blue: BD-B; black star indicates individuals with qi > 0.2, for clusters specific to commercial lines (STRUCTURE analysis for K = 6). [file 1471-2156-13-34-S11.pdf]

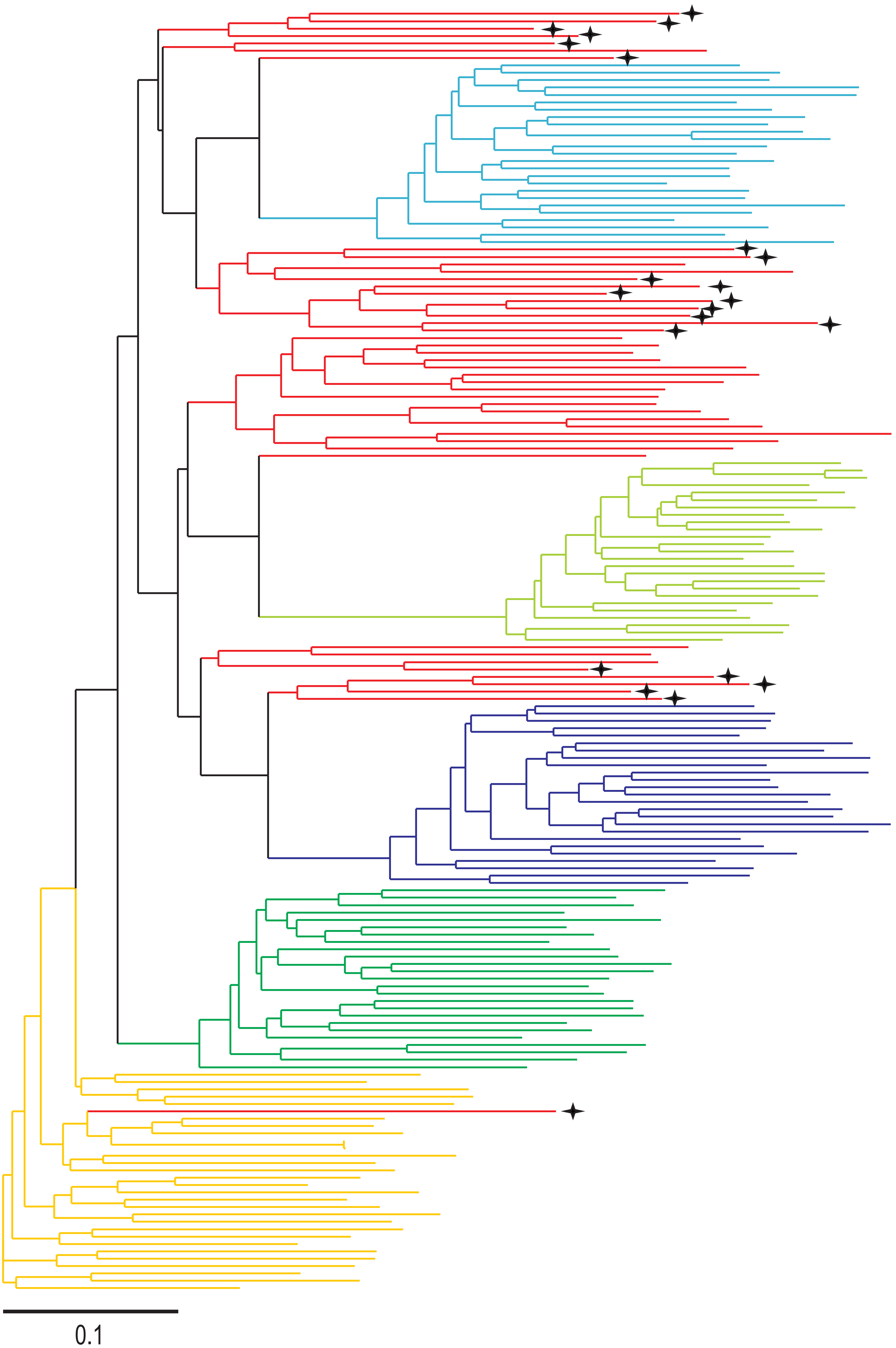

Additional file 11 – Individual neighbor-joining dendrogram based on *DAS* distance among 175 samples representing local chickens from Morocco (n=46) and 5 commercial lines (n=129). red: Morocco; orange: BS-D; dark green: BS-C; dark blue: Bel-C; light green: Wel-A; light blue: BD-B; black star indicates individuals with $q_i > 0.2$, for clusters specific to commercial lines (STRUCTURE analysis for K=6).
